# In-hospital outcomes among older medical inpatients admitted to aged care wards after activation of a 2-tier rapid response system

**DOI:** 10.1186/s12877-023-03947-6

**Published:** 2023-07-11

**Authors:** David Basic, Danielle Ní Chróinín

**Affiliations:** 1 Department of Geriatric Medicine, Liverpool Hospital, Locked Mail Bag 7103, Liverpool BC, NSW 1871 Australia; 2 Department of Geriatric Medicine, Liverpool Hospital, and South Western Sydney Clinical School, University of New South Wales, Sydney, NSW Australia

**Keywords:** Rapid response team, Aged, Inpatients, Outcome assessment

## Abstract

**Background:**

The outcomes of rapid response systems (RRS) are poorly established in older people. We examined the outcomes in older inpatients at a tertiary hospital that uses a 2-tier RRS, including the outcomes of each tier.

**Methods:**

The 2-tier RRS comprised the clinical review call (CRC) (tier one) and the medical emergency team call (MET) (tier two). We compared the outcomes in four configurations of MET and CRC (MET with CRC; MET without CRC; CRC without MET; neither MET nor CRC). The primary outcome was in-hospital death, and secondary outcomes were length of stay (LOS) and new residential facility placement. Statistical analyses were carried out using Fisher’s exact tests, Kruskal-Wallis tests, and logistic regression.

**Results:**

A total of 433 METs and 1,395 CRCs occurred among 3,910 consecutive admissions of mean age 84 years. The effect of a MET on death was unaffected by the occurrence of a CRC. The rates of death for MET ± CRC, and CRC without MET, were 30.5% and 18.5%, respectively. Patients having one or more MET ± CRC (adjusted odds ratio [aOR] 4.04, 95% confidence interval [CI] 2.96–5.52), and those having one or more CRC without MET (aOR 2.22, 95% CI 1.68–2.93), were more likely to die in adjusted analysis. Patients who required a MET ± CRC were more likely to be placed in a high-care residential facility (aOR 1.52, 95% CI 1.03–2.24), as were patients who required a CRC without MET (aOR 1.61, 95% CI 1.22–2.14). The LOS of patients who required a MET ± CRC, and CRC without MET, was longer than that of patients who required neither (*P* < 0.001).

**Conclusions:**

Both MET and CRC were associated with increased likelihood of death and new residential facility placement, after adjusting for factors such as age, comorbidity, and frailty. These data are important for patient prognostication, discussions on goals of care, and discharge planning. The high death rate of patients requiring a CRC (without a MET) has not been previously reported, and may suggest that CRCs among older inpatients should be expediated and attended by senior medical personnel.

## Background

Medical emergency teams, also known as rapid response teams (RRTs), were first implemented at Liverpool Hospital in Sydney, Australia, in 1990 [1]. The rationale for their use followed the observation that many unexpected deaths and cardiac arrests are preceded by physiological instability [2]. In an effort to prevent such adverse outcomes, RRTs (or rapid response systems [RRS]) are used in many countries to manage and prevent physiologic deteriorations among medically unwell patients [3]. Despite ongoing controversy on the effectiveness of RRS [4, 5], there is evidence that they reduce in-hospital mortality and cardiopulmonary arrests in the adult population [6].

There is considerable variation in the activation criteria, structure, composition, and leadership of RRS [7–10]. Graded RRS have been implemented in Australia, the United Kingdom and Ireland as best practice [4]. Whereas the United Kingdom uses three levels of response (low, medium and high) [4, 11], 2-tier RRS are commonly used in Australia [5, 9, 12, 13] and elsewhere [14]. A 2-tier RRS with standard calling criteria and standard observation charts (known as ‘Between the Flags’) was established by the Clinical Excellence Commission [15] and implemented in 2010 in over 225 New South Wales (Australia) hospitals. In order to balance clinical effectiveness and limited intensive care resources, similarly structured RRS have been implemented elsewhere in Australia [9, 12, 13, 16] and overseas [14]. The primary goals of the first tier are to recognise and correct early physiological instability, identify patients unlikely to benefit from aggressive medical therapy, and reduce the number of more urgent calls. The main goal of the second tier is to respond to appropriate patients who fail to improve with first-tier interventions [14]. Tier activation, however, does not necessarily follow this order (e.g., immediate second tier activation for cardiac or respiratory arrests, or serious concern by staff). A 2-tier response to clinical deterioration has been shown to reduce overall intensive care unit (ICU) mortality, and ICU admissions triggered by non-cardiorespiratory criteria [13]. High levels of satisfaction by clinical staff have also been reported [9].

Although multitiered RRS are not a new concept, there is considerable heterogeneity in patient and RRS characteristics of such teams [9, 12–14, 16], making it difficult to evaluate the outcomes of patients requiring this intervention. Furthermore, the utilisation and outcomes of multitiered RRS are poorly established in older populations. These factors are important for patient prognostication, hospital and critical care resource planning, and discharge planning. In this study we evaluated the outcomes of a 2-tier RRS among older inpatients, as well as variables associated with in-hospital death.

## Methods

### Study location and study participants

This retrospective cohort study was conducted at a tertiary hospital in Sydney, Australia. Study participants were consecutive patients aged ≥ 65 years discharged between 1 January 2013 and 30 September 2015 from two adjacent 25-bed acute aged care wards (ACW). Criteria for admission to the acute ACW were broad and included age-associated conditions such as delirium, dementia with behavioural problems, malnutrition associated with age-related conditions such as dementia and frailty, deconditioning and/or functional decline, gait abnormality, recent falls, and uncomplicated fragility fractures (e.g., pelvic, vertebral, humeral, rib). Patients were categorised according to whether they had a rapid response call. The study was approved by the Local Health District Human Research Ethics Committee.

### Medical emergency team and clinical review activation criteria

The 2-tier RRS operated 24/7 and comprised the clinical review call (CRC) (tier one response) and the medical emergency team call (MET) (tier two response). The activation criteria for both are shown in Table 1. In New South Wales, the MET and CRC activation criteria have not changed substantially for more than 10 years [5, 17]. Many RRS in Australia and in other countries use similar activation criteria, though the number of criteria and thresholds for heart rate, blood pressure, respiratory rate and mental state may vary [14, 18]. Whereas the MET was attended by an intensive care registrar, a medical registrar and an intensive care nurse, most CRCs were attended by a single junior doctor. This disparity in attending personnel between the MET and the CRC was based on the observation that most RRS interventions do not require the presence of senior staff [6]. Intensive care registrars and medical registrars were doctors with at least two years of hospital experience, and who had started a specialty training program (either uncoupled to a particular specialty or undertaking higher training in a chosen specialty). The response time for a MET was immediate, with staff at the bedside within several minutes. Although the junior doctor was expected to attend a CRC as soon as possible, the time frame for attendance was within 30 min.

**Table 1:** Medical emergency team and clinical review activation criteria^a^

Medical emergency team criteria^b^	Clinical review criteria^c^
Respiratory rate < 5 or > 30 breaths/min	Respiratory rate 5–10 or 25–30 breaths/min
Oxygen saturation < 90%	Oxygen saturation 90–95%
Systolic blood pressure < 90 or > 200 mmHg	Systolic blood pressure 90–100 or 180–200 mmHg
Heart rate < 40 or > 140 beats/min	Heart rate 40–50 or 120–140 beats/min
↑ Oxygen requirement to maintain saturation > 90%	↑ Oxygen requirement
Cardiac or respiratory arrest	Temperature < 35.5^o^C or > 38.5^o^C
Airway obstruction or stridor	↓ LOC or new onset of confusion
Unresponsive or sudden ↓ LOC or ↓ GCS ≥ 2 points	↓ From A to V on AVPU scale
Only responds to P on AVPU scale	Poor peripheral circulation
Seizures	Excess or increasing blood loss
Deterioration not reversed within 1 h of clinical review	Urine output < 100mL/4 h or < 0.5 mL/kg/h for 4 h (via IDC)
ABG: PaO_2_ < 60 or PaCO_2_ > 60 or pH < 7.2 or BE < − 5	Polyuria, urine > 200mL/h/2 h (in absence of diuretic)
VBG: PvCO_2_ > 65 or pH < 7.2	Greater than expected fluid loss from a drain
Blood glucose < 4 mmol/L or > 20 mmol/L with ↓ LOC	New, increasing, or uncontrolled pain (including chest pain)
Lactate ≥ 4 mmol/L	Blood glucose < 4 mmol/L or > 20 mmol/L with no ↓ LOC
Urine output < 200mL/8 h or < 0.5 mL/kg/h for 8 h (via IDC)	Ketonaemia > 1.5 mmol/L or ketonuria 2 + or more
Serious concern by staff	Concern by staff
Serious concern by any patient or family member	Concern by any patient or family member

ABG = arterial blood gas. AVPU = alert, voice, pain, unresponsive scale. BE = base excess. GCS = Glasgow coma scale. IDC = indwelling catheter. LOC = level of consciousness. VBG = venous blood gas.

^a^Based on NSW Health Standard Calling criteria. [15]

^b^The medical emergency team includes an intensive care unit registrar, a medical registrar, and an intensive care unit nurse.

^c^Clinical reviews are mostly attended by a junior medical officer.

### Measures

Sociodemographic information included the preadmission domicile, the ability to speak English (able to provide a medical history without an interpreter) and the country of birth. The country of birth was dichotomised according to whether English was the predominant spoken language. Patients from non-English-speaking backgrounds (NESB) were born in countries other than Australia, New Zealand, United States of America, Canada, South Africa, Great Britain, and Ireland [42]. The Canadian Study of Health and Aging Clinical Frailty Scale (CSHA-CFS) [19], a simple 7-category ordinal scale, was used to measure the degree of frailty present one month before admission, with higher categories indicating increasing frailty. Up to 15 medical diagnoses were coded per patient, and were based on version 5.1 of the Australian Refined Diagnosis Related Groups classification system [20]. In-hospital death, length of stay (LOS) and discharge domicile were also documented.

Up to five METs and CRCs were recorded per patient. Data on the former included the date and time, whether transferred to the ACW within the previous 24 h, the predominant reason for the call, and the outcome (death, admission to the ICU). Data on CRCs included only their date and time. Normal working hours were defined as those occurring between 0830 and 1700 h.

### Outcome measures

The primary outcome of interest was in-hospital death. Secondary outcomes were LOS and new residential facility placement. Length of stay was defined as the number of days between arrival on the ACW and discharge from the hospital. Patients admitted to and discharged from the ACW on the same day were allocated a LOS of 0.5 days. Low-care residential facilities were those that provided meals, laundry services, help with personal care, and occasional nursing care, whereas high-care residential facilities were those that provided complete, or almost complete, assistance with most daily living activities.

### Statistical analyses

We calculated the rate of death in four patient groups or configurations of MET and CRC (MET with CRC; MET without CRC; CRC without MET; neither MET nor CRC). The rates of death determined which groups were subsequently evaluated in multivariate logistic regression models. We decided, a priori, to combine groups that had similar death rates for evaluation in regression models. We hypothesised that once a MET occurred, a CRC would have little additional impact. The association between rapid response calls and new residential facility placement was also evaluated, as was the association with LOS. The covariates considered for inclusion in the regression models were based on the literature [21–24] and on biological plausibility. Independent predictors (*P* < 0.05) from our dataset were included in the final models, together with the study variables (MET and CRC occurrences). In the regression models, MET and CRC refer to one or more occurrences of MET and CRC, respectively, per patient. To simplify interpretation, frailty was dichotomised (moderately or severely frail [categories 6–7] vs. other [categories 1–5]). Between-groups associations were evaluated using Fisher’s exact tests for dichotomous variable and Kruskal-Wallis tests for ordinal variables. Analyses were performed using SAS, version 9.4 (SAS Institute, Inc., Cary, NC).

We report our findings as per the Strengthening the Reporting of Observational Studies in Epidemiology (STROBE) guidelines [43].

## Results

### Characteristics of participants

The mean age of all 3,910 patients was 84.1 ± 7.2 years and 93.5% were admitted through the emergency department. Most patients (67.6%) resided in the community before admission. Among those born in NESB countries (59.0%), 41.4% were able to speak English. Table 2 shows the characteristics of the study participants by MET and CRC configurations. A diagnosis of dementia was common in the study population (Table 2). However, very few patients were admitted for dementia as a stand-alone diagnosis, and those that were almost always had behavioural and psychological symptoms of dementia severe enough to warrant admission. Most patients with dementia had delirium and/or an acute condition documented during their hospital stay.

**Table 2:** Characteristics of study patients by MET and CRC configurations

Characteristic	MET with CRC (n = 341)	MET without CRC (n = 154)	CRC without MET (n = 639)	Neither MET nor CRC (n = 2930)
Age (years)	84.4 ± 7.0	85.0 ± 6.9	84.6 ± 7.1	84.0 ± 7.3
Male (%)	44.0	39.6	41.5	41.0
NESB country of birth (%)^ab^	61.6	61.7	59.3	58.6
Able to speak English (%)	63.1	64.3	66.0	65.5
Preadmission residence (%)				
Community	67.7	66.2	66.5	67.8
HC-RACF	26.7	30.5	26.5	25.5
LC-RACF	5.6	3.3	7.0	6.7
Referral source (%)				
Emergency department	92.1	93.5	93.7	93.6
Consult and transfer of care	7.6	5.8	6.1	6.2
Other^c^	0.3	0.7	0.2	0.2
CSHA-CFS category (%)^bd^				
Moderate-severe (categories 6–7)	81.5	83.7	79.4	69.7
Other (categories 1–5)	18.5	16.3	20.6	30.3
Medical diagnosis (%)^be^				
Dementia	46.8	47.2	46.6	47.2
Delirium	57.8	51.4	53.5	43.5
Malignant neoplasm (any)	12.7	12.7	12.4	10.5
Fracture (any)	10.7	11.3	10.1	12.9
Acute myocardial infarction	11.7	12.0	8.3	4.8
Cardiac failure	35.1	33.8	23.4	15.9
Deconditioning	31.5	31.0	31.9	26.8
Malnutrition (severe)	17.9	14.8	17.9	10.2
Renal failure (acute)	33.1	27.5	29.4	17.3
Respiratory infection	51.0	40.9	40.9	25.9
Septic shock	14.6	11.3	10.6	5.3
COPD	14.0	11.3	10.6	10.2
Type 2 respiratory failure	10.4	10.6	4.6	2.2
Stroke/ intracranial haemorrhage	10.1	6.3	8.7	9.4
Diabetes	18.8	20.4	26.0	22.9

COPD = chronic obstructive pulmonary disease. CRC = clinical review call. CSHA-CFS = Canadian Study of Health and Aging Clinical Frailty Scale. HC-RACF = high-care residential aged care facility. LC-RACF = low-care residential aged care facility. MET = medical emergency team call. NESB = non-English-speaking background.

^a^NESB country of birth data were missing for 3 (0.1%) patients.

^b^Percentages refer to patients with non-missing data.

^c^Other refers to direct admission from the community, clinics, and other hospitals.

^d^CSHA-CFS category data were missing for 464 (11.9%) patients.

^e^Medical data were missing for 469 (12.0%) patients.

### Rapid response calls

A total of 433 METs occurred on the ACW during the study period, with 275 patients having a single MET. The number of patients having two, three, four and five METs were 50, 10, 2 and 4, respectively. More METs occurred outside of normal working hours then during working hours (53.3% vs. 46.7%). Among 89 patients transferred to the ACW within 24 h before a MET, 69 (77.5%) came from the emergency department, 18 (20.2%) came from another hospital ward, and two (2.3%) came from the ICU. The most common reasons for a MET were hypotension (n = 103), sudden decrease in level of consciousness (n = 99), high respiratory rate (n = 53), hypertension (n = 49) and reduced oxygen saturation (n = 41). Sixteen patients had a cardio-respiratory arrest, 14 of whom died in hospital (including the two transferred to the ICU). One of the two survivors was discharged to a private home, while the other needed placement in a high-care residential aged care facility.

Thirty-two (7.4%) patients were transferred to the 60-bed ICU after a MET, 93.8% of whom resided in the community. Among the 119 patients from a residential aged care facility who had a MET, the rate of transfer to ICU was 1.7%. Eleven (34.4%) of 32 patients transferred to the ICU died.

A total of 1,395 CRCs occurred during the study period. The number of patients having one, two, three, four and five CRCs were 501, 186, 72, 29 and 38, respectively. The first MET was preceded by at least one CRC on 180 occasions (41.6%), with 66 of these occurring on the same day as the MET (median time 61 min before the MET, interquartile range [IQR] 18–216 min). More CRCs occurred outside of normal working hours (56.1% vs. 43.9%).

Patients diagnosed with delirium, septic shock, respiratory infection, type 2 respiratory failure, acute renal failure, acute myocardial infarction, cardiac failure and malnutrition were more likely to have a rapid response call (MET and/or CRC) than those without these diagnoses (Table 2).

### In-hospital deaths and death rates

A total of 360 (9.2%) patients died in the hospital. Table 3 shows the rates of death by MET and CRC configurations. The effect of a MET on death was unaffected by the occurrence of a CRC, whereas the rate of death was lower when a CRC was activated without a preceding or subsequent MET. The rates of death for MET ± CRC, and CRC without MET, were 30.5% and 18.5%, respectively. These rates were considerably higher than the rate (4.7%) when neither a MET nor a CRC occurred (Table 3). Patients transferred to the ACW within 24 h before a MET were not at higher risk of death than others (*P* = 0.50). Patients who had five CRCs had a death rate of 50.0%.

**Table 3:** In-hospital deaths by MET and CRC combinations

Died	Group 1 (n = 341)	Group 2 (n = 154)	Group 3 (n = 639)	Group 4 (n = 2930)
	MET with CRC^a^	MET without CRC	CRC without MET	Neither
Yes (n., %)	104 (30.5)	45 (29.2)	118 (18.5)	138 (4.7)
No (n., %)	237 (69.5)	109 (70.8)	521 (81.5)	2792 (95.3)

Note: MET and CRC data refer to one or more reviews.

CRC = clinical review call. MET = medical emergency team call.

^a^MET with a preceding or subsequent CRC.

Table 4 shows the unadjusted and adjusted odds ratios for in-hospital death. The configurations MET with CRC, and MET without CRC, are shown together (MET ± CRC). Patients having one or more MET ± CRC (adjusted odds ratio [aOR] 4.04, 95% confidence interval [CI] 2.96–5.52, *P* < 0.001), and those having one or more CRC without a MET (aOR 2.22, 95% CI 1.68–2.93, *P* < 0.001) were significantly more likely to die, after adjusting for age, frailty, malnutrition, acute renal failure, respiratory infection, septic shock, type 2 respiratory failure, and stroke/intracranial haemorrhage. Each of these variables was also independently associated with risk of in-hospital death (Table 4).

**Table 4:** Adjusted and unadjusted odds ratio estimates for in-hospital deaths

Variable^a^	Unadjusted OR (95% CI)^b^	MET ± CRC aOR (95% CI)^cd^	CRC aOR (95% CI)^ce^
MET ± CRC	5.68 (4.37–7.39)	4.04 (2.96–5.52)	
CRC without MET	2.84 (2.23–3.60)		2.22 (1.68–2.93)
Age (years, per unit increase)	1.04 (1.02–1.06)	1.03 (1.01–1.05)	1.03 (1.01–1.05)
Frailty^f^	4.58 (3.10–6.74)	2.90 (1.91–4.40)	2.76 (1.83–4.15)
Malnutrition	2.87 (2.20–3.74)	1.87 (1.37–2.55)	1.80 (1.33–2.45)
Acute renal failure	3.70 (2.94–4.65)	2.32 (1.78–3.03)	2.35 (1.81–3.06)
Respiratory infection	3.48 (2.78–4.37)	2.38 (1.84–3.08)	2.52 (1.96–3.25)
Septic shock	7.99 (6.00–10.65)	5.79 (4.15–8.07)	6.00 (4.34–8.32)
Type 2 respiratory failure	5.89 (3.97–8.76)	3.72 (2.35–5.89)	4.60 (2.95–7.17)
Stroke/intracranial haemorrhage	1.90 (1.38–2.60)	2.17 (1.51–3.12)	2.23 (1.55–3.19)

CI = confidence interval. CRC = clinical review call. MET = medical emergency team call. aOR = adjusted odds ratio.

^a^Medical or frailty data were missing in 500 (12.8%) patients.

^b^The effect of each variable was evaluated in isolation.

^c^341 deaths were modeled.

^d^The effect of MET, with or without preceding or subsequent CRC, was adjusted for the covariates listed in the column.

^e^The effect of CRC, without preceding or subsequent MET, was adjusted for the covariates listed in the column.

^f^Frailty was dichotomised as moderately or severely frail vs. other.

### Length of stay and new residential facility placement

Although the LOS for MET ± CRC (median 14 days, IQR 7–24) was longer than the LOS for CRC without MET (median 12 days, IQR 7–21), this difference was not significant (*P* = 0.10). However, these LOS were considerably longer than the LOS among patients who had neither a MET nor a CRC (median 7 days, IQR 4–13, *P* < 0.001) (Table 5). Patients who had five CRCs (n = 38) had a median LOS of 30.5 days (IQR 18–41). Among those who were discharged alive (n = 19), 10 (52.6%) were newly placed in a high-care residential aged care facility.

**Table 5:** Length of stay and change in discharge domicile

Characteristic	All patients (n = 3910)	MET ± CRC^a^ (n = 341)	CRC^b^ (n = 639)	Neither (n = 2930)
LOS (median, IQR)	8 (4–15)	14 (7–24)	12 (7–21)	7 (4–13)
Domicile change				
HC-RACF (n., %) ^c^	425, 12.0	40, 16.9	83, 15.9	302, 10.8
LOS (median, IQR)	17, 11–27	27, 15.5–43	21, 16–33	16, 10–24
LC-RACF (n., %) ^c^	98, 2.8	8, 3.4	13, 2.5	77, 2.8

CRC = clinical review call. HC-RACF = high-care residential aged care facility. IQR = interquartile range. LC-RACF = low-care residential aged care facility. LOS = length of stay. MET = medical emergency team call.

^a^One or more MET, with or without preceding or subsequent CRC.

^b^One or more CRC, without preceding or subsequent MET.

^c^Numbers and percentages refer to patients who were discharged alive (360 patients died before discharge).

A total of 425 patients were newly placed in a high-care residential facility. Of these, 123 had a RRS review before placement. Their LOS (median 23 days, IQR 16–36) was significantly longer than patients who did not need a RRS review before placement (n = 302, median 16 days, IQR 10–24) (*P* < 0.001). Both MET ± CRC, and CRC without MET, also had significantly longer LOS before placement, compared with patients who did not need a RRS review (both *P* < 0.001). Their LOS are shown in Table 5. Similar data for low-care residential facility placement were not calculated due to low numbers of patients needing this care.

On adjusted analysis, patients who required a MET call (± CRC) were more likely to be placed in a high-care residential facility (aOR 1.52, 95% CI 1.03–2.24, *P* = 0.03), after adjusting for age, dementia, behavioural and psychological symptoms of dementia and frailty. Patients who required a CRC without a MET were also more likely to be placed (aOR 1.61, 95% CI 1.22–2.14, *P* < 0.001). Low-care residential facility placement was not evaluated because few patients needed this level of care.

## Discussion

In this large study of almost 4,000 aged care patients with high rates of medical morbidity and frailty, both MET and CRC were associated with increased likelihood of death. This association persisted even after adjusting for factors such as age, comorbidity and frailty. Almost one in three patients who had a MET died in the hospital. Reported in-hospital mortality rates of patients requiring rapid response system (RRS) activation in other studies [14, 25] were similar to our patients who required a MET. A Canadian study [25] reported an in-hospital death rate of 36.2% among patients ≥ 75 years of age after activation of a single-tier RRS. A Singaporean study [14] of younger patients (mean age 64 years) found a similar death rate (34.9%) in patients who required second-tier RRT activations. The rate of death of patients requiring a CRC without a MET was also high, a finding that has not been previously reported. These data are important because they help with patient prognostication, discussions on goals of care with patients and their families, and triage decisions (including transfers of patients to the ICU). Our finding that more than 40% of METs were preceded by a CRC, with many on the same day, underscores the importance of the CRC. This may suggest that CRCs among older patients should be expediated and attended by more senior medical personnel or even a dedicated team. It is unknown, however, whether these interventions would reduce avoidable deaths, while at the same time increasing the burden on medical staff, disrupting normal hospital routines, and impacting on other aspects of care [16].

Our findings show that patients who had a MET call for a cardio-respiratory arrest had a particularly poor prognosis. Although age should not be a stand-alone criterion for withholding cardiopulmonary resuscitation (CPR) after a cardio-respiratory arrest, it is important that patients and their families have a realistic and early understanding of CPR and its outcomes, including the survival rates, the potential impacts on function, and the possibility that survival merely postpones inevitable death, especially in situations where older patients have serious underlying comorbidities [44]. There are many opportunities to revisit plans for resuscitation and other goals of care after CRCs, given the relatively high frequency of CRCs, many of which occur before the first MET, and the high in-hospital death rates associated with CRCs.

The need for a RRS review was also associated with increased hospital LOS, and likelihood of transfer to a high-care residential facility. MET, and CRC without MET, significantly increased the LOS of patients discharged to a high-care residential facility. These findings are important for service planning, both during the hospital stay and after discharge. They should also help to inform patients and their carers on goals of care and likely outcomes following acute illness.

We identified a number of factors independently associated with increased risk of death, including some which may be modifiable, such as malnutrition and frailty, both of which were criteria for admission to our units. While it is debatable whether premorbid frailty could be modified during an acute hospital stay to an extent that it reduced avoidable deaths, there is evidence that malnutrition is a major risk factor for frailty [26] and death [27], and that malnutrition interventions reduce in-hospital mortality compared with usual care among hospitalised adults [28]. Although dementia was not identified as a risk factor for death in our cohort, we note that malnutrition may also be a marker of advancing dementia [45]. The consequences of malnutrition, which are more severe in older people, are many and include increased risk of infection, muscle wasting and reduced muscle strength, fall and fracture risk, poor wound healing, and impaired recovery from acute illness [26], many of which are associated with increased hospital mortality. While variables such as acute renal failure, sepsis and respiratory failure have been identified as risk factors for poor outcomes in the context of emergency response systems, others such as malnutrition have often been overlooked [29–31]. The reasons for its absence from the RRS-related literature might include under-recognition, failure to explore malnutrition as a variable, and/or because it is more common in older populations compared to the younger cohorts often described in the RRS-related papers [27, 32].

Strengths of our paper include the large study population, the comprehensive and standardised data collection, the adjustment for important variables, and the inclusion of all consecutive patients admitted to the ACW. As such, our findings reflect real-world outcomes in older patients presenting with geriatric syndromes and other age-related disorders. We also captured information regarding residential facility placement, an outcome likely to be of interest to healthcare providers, patients and carers.

We note that our study focussed on outcomes in the setting of a 2-tier RRS. The potential benefits of involvement of the ward team (tier one response) include continuity of care, avoidance of de-skilling, the early identification of patients unlikely to benefit from aggressive interventions, and a more efficient use of hospital and critical care resources [14, 16]. However, a 2-tier RRS may be associated with a delay in response call activation and intervention, where a delay of more than 15 min has been shown to significantly increase in-hospital mortality and LOS [33]. The 2-tier RRS is not a universal system, and the composition of RRTs, and criteria for review, vary between settings and papers [9, 10], and are likely ‘influenced by available expertise, patient case-mix and resources in each hospital’ [14]. While the need for RRS has been embraced in Australia and in other jurisdictions [11, 34], the make-up of these has not been standardised even at a national level [35].

Our study has several limitations. We have no data on the goals of care of patients’ and carers, and whether these changed after RRS activation. We did not measure the times to first tier (CRC) and second tier (MET) interventions, or the reasons for CRC activation. It is possible that delayed responses to CRCs may have resulted in avoidable deaths, thus overestimating their impact. If CRCs and METs serve as a prompt to re-evaluate the likely prognosis and the goals of care, then this may increase the apparent risk of death following a rapid response review. We acknowledge that all death is not ‘inappropriate’, and that death is not necessarily the outcome most feared by patients [36]. As this was a single-hospital study, the generalisability of our findings is limited, particularly as almost 60% of patients were born in NESB countries and a substantial proportion were unable to speak English. Furthermore, many of our patients were admitted based on age-related conditions and syndromes (e.g., delirium) and all were managed in one of two aged care wards, with many having illnesses of low to moderate acuity. The population of older people is larger and more diverse than our study population, and our results cannot be extrapolated to all older inpatients, including surgical inpatients, medical inpatients managed outside of dedicated aged care wards, and patients with illnesses of high acuity. Because beliefs regarding treatment, and issues related to death and dying may differ across NESB populations [37], interpreting the impact of METs and CRCs on death is complex, particularly when there are linguistic difficulties. Although our data derive from 2013 to 2015, the 2-tier RRS in our hospital has largely remained unchanged since the inception of this study, and we believe that comorbidities among older inpatients have not changed substantially between then and now. However, it is possible that issues such as advance care planning and resuscitation discussions may have improved, especially in the context of the COVID-19 pandemic and associated concerns regarding prognosis and resource availability [38–40]. Increases in advance care planning may have impacted on decisions regarding ceilings of care and the focus of treatment.

A substantial number of patients had multiple CRCs. The outcomes of the 38 patients who had five CRCs were especially poor, with most dying in the hospital or needing new placement in a high-care residential aged care facility. Possible explanations for the need for multiple CRCs include suboptimal communication between the outside of normal working hours CRC responder and the ward team, the lack of appreciation of the importance of CRCs as a prognostic marker, the failure to re-evaluate plans for resuscitation and other goals of care after CRCs, and the overlap between some of the admission criteria and independent predictors of CRCs (e.g., malnutrition and frailty).

Further studies could focus on optimising RRS to improve patient care and outcomes, exploring and understanding the ‘consumer’ perspective, and ensuring that open and honest discussions about goals of care and likely prognosis are embedded in clinical practice [41].

## Conclusions

This study shows that METs and CRCs are common among older people admitted to an acute aged care ward, and that they are associated with increased in-hospital death rates, LOS, and new high-care residential facility placements. These findings are important for discussions on prognosis and goals of care, triage decisions, hospital and critical care resource planning, and discharge planning. The high death rate of patients requiring a CRC (without a MET) may suggest that the 30-minute time frame for CRC attendance at our hospital is too long. The identification of malnutrition as an independent risk-factor for in-hospital death is important because malnutrition is potentially modifiable.

## Data Availability

The datasets used and/or analysed during the current study are available from the corresponding author on reasonable request.

## Abbreviations

ACW: Aged care wards
aOR: Adjusted odds ratio
CI: Confidence interval
CPR: Cardiopulmonary resuscitation
CRC: Clinical review call
CSHA-CFS: Canadian Study of Health and Aging Clinical Frailty Scale
ICU: Intensive care unit
IQR: Interquartile range
LOS: Length of stay
MET: Medical emergency team call
NESB: Non-English speaking background
RRS: Rapid response system
RRT: Rapid response team.
